# Sphingosine 1-Phosphate Signaling and Its Pharmacological Modulation in Allogeneic Hematopoietic Stem Cell Transplantation

**DOI:** 10.3390/ijms18102027

**Published:** 2017-09-21

**Authors:** Philip Smith, Catherine O’Sullivan, Peter Gergely

**Affiliations:** 1 Novartis Institutes for BioMedical Research, WSJ-386, CH-4002 Basel, Switzerland; philip.smith@novartis.com (P.S.); osullc12@tcd.ie (C.O.); 2 School of Medicine, Trinity Biomedical Sciences Institute, Trinity College Dublin, 152-160 Pearse Street, Dublin 2, Ireland

**Keywords:** sphingosine 1-phosphate, haemopoietic stem cell transplantation, graft-versus-host disease, graft versus leukaemia

## Abstract

Allogeneic haemopoietic stem cell transplantation (HSCT) is increasingly used to treat haematological malignant diseases via the graft-versus-leukaemia (GvL) or graft-versus-tumour effects. Although improvements in infectious disease prophylaxis, immunosuppressive treatments, supportive care, and molecular based tissue typing have contributed to enhanced outcomes, acute graft-versus-host disease and other transplant related complications still contribute to high mortality and significantly limit the more widespread use of HSCT. Sphingosine 1-phosphate (S1P) is a zwitterionic lysophospholipid that has been implicated as a crucial signaling regulator in many physiological and pathophysiological processes including multiple cell types such as macrophages, dendritic cells, T cells, T regulatory cells and endothelial cells. Recent data suggested important roles for S1P signaling in engraftment, graft-versus-host disease (GvHD), GvL and other processes that occur during and after HSCT. Based on such data, pharmacological intervention via S1P modulation may have the potential to improve patient outcome by regulating GvHD and enhancing engraftment while permitting effective GvL.

## 1. Allogeneic Hematopoietic Stem Cell Transplantation (HSCT) for the Treatment of Hematological Malignances: Overview

Allogeneic hematopoietic stem cell transplantation (HSCT) is a form of immunotherapy whereby multipotent hematopoietic stem cells, derived from Human Leukocyte Antigen HLA matched donor bone marrow, peripheral blood, or umbilical cord blood are transplanted to a recipient. The recipient’s immune system is usually ablated with radiation or chemotherapy before the transplantation to eradicate the patient’s malignancy and to suppress the host immune system to enable engraftment of the donor cells. The entire adult hematopoietic system is maintained by a rare population of hematopoietic stem cells that account for ~0.01% of the total cells in the bone marrow [1]. Remarkably, infusion of a limited number of HSCs is sufficient to repopulate the entire hematopoietic system.

**Figure 1:**
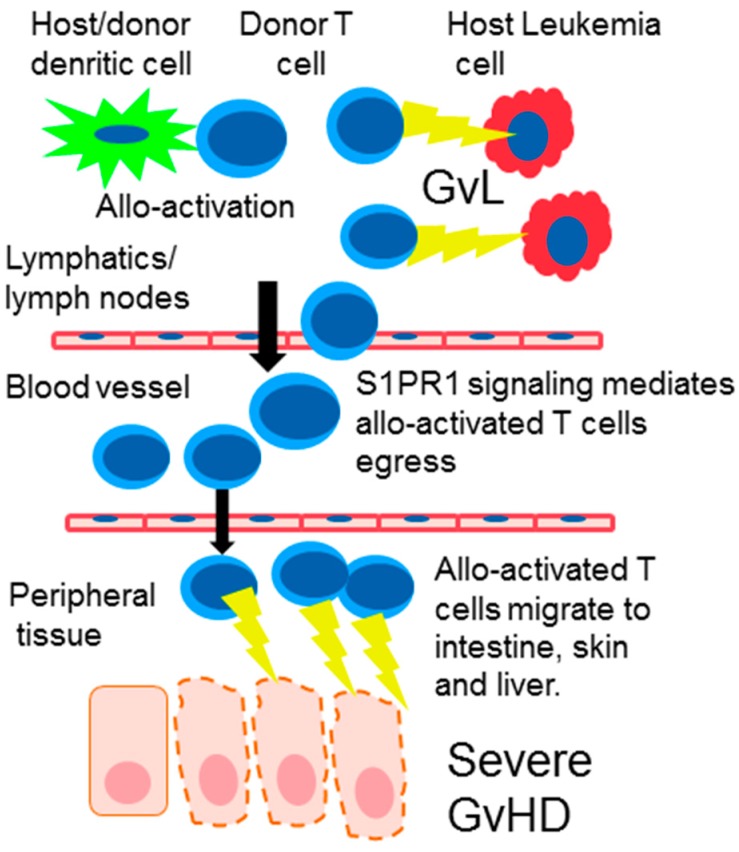
Following HSCT, donor T cells rapidly migrate to the lymphatic compartment and are allo-activated by dendritic cells. Activated T cells proliferate and orchestrate the graft-versus-leukemia (GvL) reaction killing leukemia cells. Allo-activated T cells express S1PR1 and egress from the lymph node. The allo-activated T cells then migrate to graft-versus-host disease (GvHD) target organs such as the intestine skin and liver where they instigate inflammation and tissue damage.

Allogeneic HSCT is increasingly used to treat hematological malignant diseases via the graft-versus-leukemia (GvL) or graft-versus-tumor effects in which donor lymphocytes present in the graft recognize and kill the host’s tumor cells. This procedure can be clinically very efficacious and even curative for certain haematological malignancies. However, acute graft-versus-host disease (GvHD) often arises and remains the major complication of HSCT as it is associated with high mortality, significantly limiting the more widespread use of HSCT [2]. Acute GvHD is a complex allo-immune mediated disease (as the HLA match is incomplete) primarily caused by donor T cells responding to host proteins on antigen presenting cells, resulting in clinically significant organ damage involving mainly the skin, GI tract and the liver (Figure 1).

Overall, a safer HSCT, reducing or eliminating the risk of acute GvHD, with preserved GvL would represent a major advance to treat hematological malignancies.

## 2. Sphingosine 1-Phosphate (S1P) and the S1P Receptors (S1PRs)

Sphingosine 1-phosphate (S1P) is a pleotropic lysophospholipid with zwitterionic polarity involved in numerous and diverse biological processes. S1P is derived from sphingolipid ceramide, which itself is synthesized either via the actions of sphingomyelinases in the membrane or via *de novo* pathways initiated in the endoplasmic reticulum. De-acylation of ceramide by ceramidase results in release of sphingosine ((2*S*,3*R*,4*E*)-2-amino-4-octadecen-1,3-diol), which is subsequently phosphorylated in an ATP-dependent manner by sphingosine kinases (SphK1 or SphK2) to form S1P [3]. S1P enhances cell proliferation and has pro-survival, pro-inflammatory, and pro-motility characteristic [4]. The actions of S1P are primarily mediated via S1P receptors (S1PRs). These receptors are known drug targets for multiple sclerosis, where the oral therapy Gilenya^®^ (Fingolimod, FTY720) has been approved for clinical use [5]. In vivo FTY720 is phosphorylated to the active metabolite pFTY720 via the activity of SphK2. Upon conversion pFTY720 binds to 4 of the 5 S1P receptors (S1PR1, S1PR3, S1PR4, S1PR5) with potent agonistic activity resulting in sequestration of lymphocytes into secondary lymphoid tissues [6].

Since the development of FTY720 a number of S1PR agonists and antagonists have been synthesized. Early pre-clinical work in rodents, though over simplistic, have suggested that the clinical benefits of FTY720 are attributed to lymphocyte sequestration through S1PR1 activation while the undesirable cardiac effects are due to S1PR3 binding [7,8]. These findings prompted the development of new more selective S1PR agonists with higher activity to S1PR1 compared to S1PR3. The next generation of more selective S1PR modulating compounds such as BAF312, KRP203, ozanimod, or ponesimod is now being tested in clinical trials.

## 3. Pharmacological S1PR Modulation

The mechanism of action underlying the efficacy of S1PR modulation in the attenuation of inflammatory diseases has mainly been attributed to the functional antagonistic effects of S1PR agonists at the S1PR1 subtype. Briefly, in T cells, S1P1Rs play a role in the transmigration of T cells and their egress from lymphoid tissues [9]. Agonist binding to S1PR1 results in phosphorylation of its serine rich C-terminus and subsequent internalization via β-arrestin-mediated clathrin coated vesicles. While S1P stimulation mostly results in internalization and consequent recycling of the S1PR1 back to the membrane, binding of pFTY720 to S1PR1 leads to sustained internalization of the receptor with subsequent polyubiquitinylation and degradation [10,11,12]. Despite internalization it has been demonstrated that pFTY720 when bound to S1PR1 results in specific receptor conformation that allows persistent signaling [13]. Continued signaling of internalized pFTY720-bound S1PR1 resulted in enhanced chemotactic migration of endothelial cells which may translate in vivo to decreased endothelial barrier permeability and thus contribute to reduced transmigration of T cells [13]. In summary, the binding of agonistic compounds such as pFTY720, BAF312 or KRP203 to the S1PR1 subtype on T lymphocytes results in internalization of the S1PR1 rendering the T cells unresponsive to egress signals from S1P. Thus the T cells are ″sequestered″ in the lymph nodes and are no longer able to recirculate to peripheral tissues [14,15,16].

## 4. S1PR Modulation in Graft-Versus-Host Disease (GvHD)

GvHD is a serious and potentially fatal complication following allogeneic HSCT. The disease is divided into acute and chronic forms which were historically defined by time of onset, with acute GvHD occurring within the first 100 days and chronic GvHD occurring more than 100 days after the transplantation. However, more recently acute and chronic GvHD are diagnosed based on clinical manifestations [17].

Much of our understanding of the pathophysiology of GVHD has developed via the utilization of murine models and while there are many caveats in translating directly from bench to bed side the key processes that drive GvHD are common in both murine models and patients receiving HSCT [18]. The classical sequence of events that lead to GvHD begins with conditioning induced cytokine storm which activates antigen presenting cells APCs resulting in enhanced alloantigen presentation to donor T cells within secondary lymphoid tissue. Allo-activated donor T cells then expand and migrate to GVHD target tissue and promote further effector cell migration and tissue damage [19] (Figure 1).

As reviewed below, S1PR modulators have been used to demonstrate that S1P signaling plays a key role in the various pathophysiological processes involved in GvHD.

## 5. Macrophages, Dendritic Cells and T Cell Activation

Dendritic cells (DCs) play an important role in the development of GvHD by presenting allo-antigen and costimulatory signals to donor CD4^+^ and CD8^+^ cells resulting in the expansion of pathogenic allo-activated T cells and subsequent initiation of GvHD [20]. Human dendritic cells express mRNA for S1PR1, 2, 3 and 4 [21]. Furthermore FTY720 treatment of these cells induces significant reduction in S1PR1 and S1PR4 expression and shown to inhibit DC chemotaxis as well as reduce their T lymphocyte cell-stimulatory capacity [22]. In a separate study, treatment of naïve mice with FTY720 reduced the absolute number of splenic DCs by 50%. When lethally irradiated mice were transfused with allogeneic bone marrow cells and Carboxyfluorescein Diacetate Succinimidyl Ester (CSFE) labelled responder T cells FTY720 treatment reduced the number of T cells that went into cell division, which is a key step for the generation off fulminant GvHD [23].

T cells are activated via antigen presentation combined with the interaction of costimulatory molecules on the surface of T cells and APCs. T cells express Cytotoxic T-lymphocyte 4 (CTLA4) which can regulate T-cell alloreactivity by competitively binding to B7 proteins (CD80 and CD86) on APCs and hence inhibit T cell activation [24]. Interestingly, FTY720 has been reported to induce a distinct increase in CTLA4 expression which in turn leads to an up-regulation of IL-10 and TGFβ and subsequent generation of T regulatory cells [25]. In addition, CTLA4 binds protein phosphatase 2A (PP2A) which is also potently activated by the direct interference of FTY720 with SET nuclear proto-oncogene/PP2A complexes [26,27]. Therefore, upon CTLA4 ligation, PP2A can dissociate and dephosphorylate certain TCR signaling proteins and facilitate the inhibition of TCR mediated activation [28]. In addition, blockade of costimulatory molecules CD40L–CD40 was shown to be efficacious in reducing GvHD [29] and FTY720 has been shown to reduce the surface expression of CD40 on monocytes [30].

Beyond the antigen presenting capability macrophages are also key mediators of tissue inflammation and damage via the release of cytokines/chemokines and free radicals such as nitric oxide. Therefore, during GvHD tissue resident macrophages may promote conditioning-induced tissue injury via free radical release and promote inflammation via the release of chemokines that attract circulating monocytes to inflamed GvHD target organs where they differentiate into pro-inflammatory macrophages [31]. Macrophage infiltration of skin GvHD lesions has been reported to correlate directly with disease severity. In addition, dexamethasone treatment attenuated GvHD via the inhibition of macrophage functions [32]. Furthermore, macrophages and colony stimulating factor 1 (CSF-1)/CSF-1R signaling have been shown to contribute to engraftment and the development of GvHD following transplantation [33]. Importantly, blocking CSF-1/CSF-1R action by using either *Csf1r^−/−^* mice or an anti-CSF-1R antibody depleted donor macrophages from the skin and lung and resulted in a dramatic reduction in cGVHD [34]. As macrophages express S1PR1 and S1PR2 [35] regulation of monocyte/macrophage migration and/or function by S1PR modulation may be an important factor in the efficacy of S1PR agonists. Indeed the S1PR1 specific agonist VPC44116 was shown to block the secretion of pro-inflammatory cytokines from macrophages, while SEW2871 (another S1PR1 agonist) reduced tumor necrosis factor α (TNFα) and inducible nitric oxide synthase (iNOS) production and increased Arg1 expression [35] suggesting that S1PR1 modulation induces the alternative (anti-inflammatory) activation state in macrophages.

## 6. Donor T Cell Apoptosis and Egress from Lymphoid Tissue

**Figure 2:**
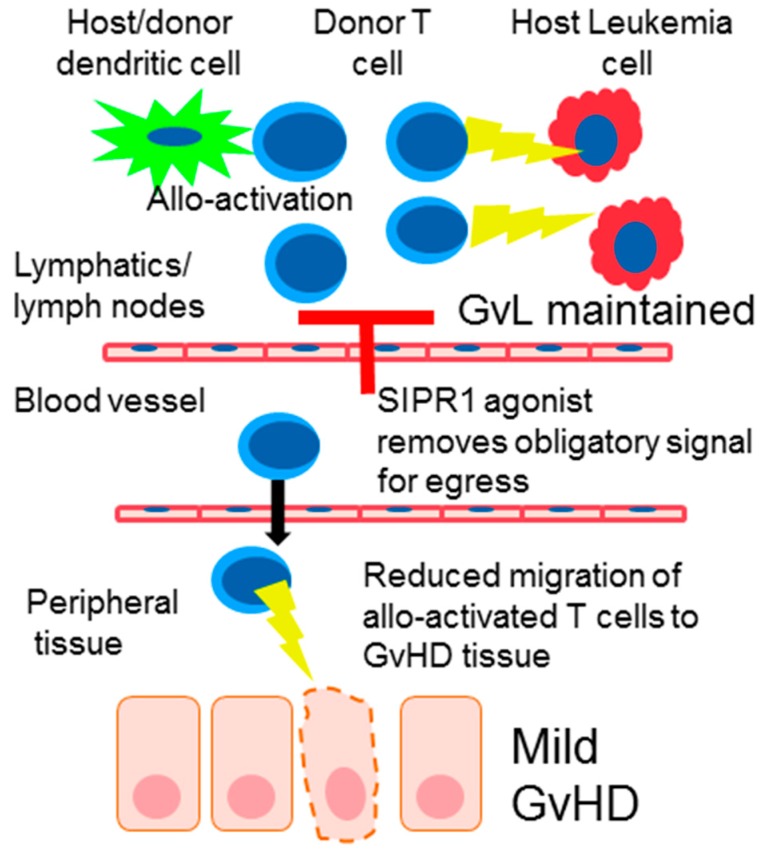
Pharmacological inhibition of S1PR signaling (denoted by red T shape) removes the obligatory signal required by activated donor T cells to egress from the lymph node. Consequently, less donor allo-activated T cells migrate to peripheral tissue resulting in reduced GvHD. However, the GvH reaction is maintained in the lymphatic system and directed towards leukemia cells resulting in effective GvL.

The principal mechanism attributed to SIP1R modulators for the treatment of inflammatory disease is the modulation of lymphocyte migration from secondary lymphoid organs to peripheral tissue. In the context of HSCT soon after donor cell infusion T cells contained in the graft migrate to secondary lymphoid tissue, in mouse models this has been show to occur within the first 72 h [36,37]. Thus, functional antagonism of SIPR1 on the surface of donor T cells in the secondary lymphoid tissue would remove the obligatory signal needed for lymphocyte egress and prevent allo-activated T cells from migrating to GvHD target tissue (Figure 2). In support of this hypothesis the reduction of GvHD by FTY720 was associated with initial trapping and accumulation of donor T cells in the lymph node on day 4. However, by day 7 the number of T cells in the lymph node had decreased due to increased activation-induced apoptosis of allo-activated donor T cells [23,38]. Interestingly FTY720 does not inhibit GvHD when it is given after HSCT thus highlighting the importance of initial modulation of donor T cell trafficking soon after HSCT [39].

In addition to activation induced apoptosis SIPR1 signaling has recently been shown to be a critical survival signal for T cells by promoting mitochondrial function [40]. Thus, loss of SIPR signaling via FTY720 may remove the mitochondrial activity resulting in in metabolic exhaustion and cell death of donor T cells [40]. Indeed a number of groups have suggested that FTY720 induced apoptosis of T cells may be an important factor underlying its efficacy [41,42,43].

## 7. The Effect of S1PR Signaling on T Regulatory Cells (Tregs) in GvHD

Much evidence exists for the beneficial functions of Tregs in the prevention of GvHD. In preclinical models, GvHD was effectively suppressed by the adoptive transfer of natural Treg cells [44,45] and Phase I clinical trials investigating the use of Tregs for the prevention of GvHD have been reported [46,47]. SIPR modulation may differentially influence Tregs and T effector cells as they these CD4^+^ sub-populations differ with respect to metabolic activity and migratory cues. Thus the effect of SIPR modulation exerted on T effector cells may have little influence on regulatory T cells. Indeed when FTY720 was co-administered with Tregs there was an additive effect with respect to inhibition of lethal GvHD demonstrating that FTY720 did not impede Treg activity [23]. There is also evidence that SIP1 signaling can directly inhibit Treg generation and survival. The proposed mechanism involves activation of S1PR1 and the subsequent activation of mTORC1 signaling. mTORC1 attenuates the activity of signal transducer Smad3, which antagonizes Treg differentiation [48]. In addition, mTORC1 also blocks suppressor of cytokine signaling 3 (SOCS3), which removes the inhibition from the signal transducer and activator of transcription 4 (STAT4) and hence promotes Th1 cell differentiation. In support of these findings functional antagonism of SIP1 signaling via FTY720 treatment was shown to increase Foxp3^+^ Tregs and promote their generation in vivo [48]. This direct effect was demonstrated in a chronic GvHD model where FTY720-mediated protection from disease was associated with an increase in the number of T regulatory cells in the spleen [49].

## 8. SIPR Signaling in Endothelial Cells

The vasculature system contributes greatly to immune function and is also now believed to play a critical part in transplant-associated rejection [50]. Vascular endothelial cells are the first host cells encountered by circulating donor T cells after allogenic stem cell transplantation [50]. Furthermore, vascular injury and arterial changes, similar to allograft vasculopathy, have been reported in aGvHD and cGvHD respectively [51,52]. Interestingly, it has been suggested that unresponsiveness to steroid therapy as seen in some GvHD cases is due to differences in endothelial vulnerability and not differences in immune activation [53,54].

Activation of vascular S1PRs with FTY720 or its analogue (R)-AFD induces the pro-survival signaling molecules, ERK and Akt which are likely involved in inhibition of endothelial cell apoptosis [55]. FTY720 is also reported to increase the survival of irradiated sinusoid endothelial cells in vitro [56]. Significant cross talk between S1PR1 and VEGF pathways have also been reported [57] and FTY720 is known to potently block VEGF-induced vascular permeability in vivo [55]. Moreover, while FTY720 may have initial cell junction tightening effects, reportedly via the translocation and assembly of β-catenin and VE-cadherin into cell-cell junctions, after repeated or high dosing, FTY720 may have barrier-disruptive effects, if the receptor is internalized [55,58]. Whether this occurs or not, however, has been contested in the literature. As treatment of the endothelium with S1P and FTY720 has been reported to lead to similar effects on barrier integrity, some have suggested that S1PR1 desensitization does not occur in the endothelium like it does in lymphocytes [55,59]. This could be explained by differential expression of the distinct post translational modification pathways required to induce internalization, recovery, or down-modulation of S1PR1 in the various cell types. In the context of GvHD the S1PR1 selective agonist, CYM5442, was shown to inhibit GvHD via a mechanism involving the reduction of macrophage infiltration at GvHD sites. The authors propose that CYM5442 may act directly on endothelial cells to increase the production of MCP-1 and MCP-3, primary chemokines important for the recruitment of macrophages from the blood, which as mentioned earlier is a key process in the development of GvHD [31].

## 9. The Effect of SIPR Signaling on the Graft vs. Leukemia Effect (GvL)

Controlling GvHD while maintaining the GvL effect is central to the success of HSCT. GvL is mediated by donor T cells and natural killer (NK) cells that are contained in the HSC graft and those cells that arise from the newly engrafted hematopoietic system. Relapse of the underlying leukemia occurs in 20–70% of patients and is influenced by various factors such as the type of leukemia, conditioning regime, donor cell source and degree of HLA matching [60,61]. The importance of GvL on relapse rate was initially demonstrated via analysis of transplant outcomes which showed that relapse rate was increased when T cells were depleted from the graft [62].

In preclinical models SIPR modulation via FTY720 inhibited GvHD pathology while at the same time permitting GvL [16,23]. While a direct effect on the leukemia was not observed the separation of GvH and GvL reactions was attributed to incomplete inhibition of the GvH reaction that permitted anti-leukemia activity but reduced peripheral tissue damage via sequestration of T cells to the secondary lymphoid compartment (Figure 2). This separation of GvH and GvL reactions with FTY720 was due to the fact that the GvH activity was maintained and restricted to the lympho-hematopoietic system where leukemia and lymphomas largely reside [16]. Another potential mechanism for an effective GvL effect with S1P modulators may arise from the differential effects observed on lymphocytes vs. NK cells. NK cells are an important immune cell population for the GvL and importantly systemic levels are not affected by FTY720 treatment [63].

Sphingolipids are potent second messengers regulating programmed cell death. Sphingosine and ceramide are pro-apoptotic which is balanced by the anti-apoptotic activity of S1P [64]. Thus S1P signaling may directly impact the GvL effect by regulating leukemia cells sensitivity to apoptosis. Various studies have shown that FTY720 has direct effects on certain leukemia growth and survival though this has been shown not to be due to S1P signaling by using FTY720 analogues that lack S1PR activity [65]. However, a recent study has shown that S1PR1 signaling enhances cell survival in naïve T cells via the modulation and mitochondrial function and metabolism. Further work is needed in order to determine the effect that functional S1P1 antagonists may have metabolic exhaustion and subsequent cell death in leukemia cells [40].

## 10. S1P and Engraftment

Timely engraftment of donor HSC is critical for safety (to reduce infection) and for efficacy (to promote GvL). The chemokine stromal cell–derived factor-1 (SDF-1) and its major receptor, CXCR4, are key players in HSC engraftment as SDF-1/CXCR4 signaling regulates migration to and from the bone marrow niche [66]. SDF-1 and S1P are reported to work synergistically to facilitate migration of primitive progenitor cells out of the bone marrow [67]. Up-regulation of S1P in immature human CD34^+^ HSCs in vitro decreases their chemotactic activity towards SDF-1 due to reduced expression of CXCR4 [68]. Interestingly, CXCR4 is reported to play a pivotal role in regulating bone marrow-derived cell engraftment and the expression levels of CXCR4 on mobilized progenitors is reported to correlate with engraftment [69]. Of note, FTY720 increased CXCR4 function in hematopoietic progenitor cells both in vitro and in vivo, thereby supporting their homing and proliferation [70]. Furthermore, pretreatment of animals and cells with FTY720 for 18 h significantly increased the number of CD34^+^CD38^−^ cells that home to the bone marrow which typically results in improved engraftment [70,71]. As S1PR1 was reported to inhibit CXCR4-dependent migration in vitro and over-expression of S1PR1 reduced CD34^+^PBPCs homing in NOD/SCID mice, it was suggested that FTY720 stimulates CXCR4 signaling in human CD34^+^PBPCs by the inactivation of S1PR1, i.e., via functional antagonism of S1PR1 [68]. In addition, FTY720 was found to phosphorylate CXCR4 which improved blood flow, however this was via the S1PR3 subtype [72]. Although the role for SDF-1-CXCR4 signaling in HSPC trafficking has been clearly demonstrated, other mechanisms independent of this pathway also play an important role in HSC migration. This is supported by observations that HSC homing and engraftment is normal or only mildly reduced when CXCR4 is pharmacologically blocked or knocked down [73,74]. Other mechanisms of HSC homing maybe particularly relevant in the context of myeloablative conditioning used for bone marrow transplantation as the highly proteolytic micro-environment in the bone marrow following conditioning leads to degradation of SDF-1 [75]. Indeed, mechanisms involving S1P that are independent of CXCR4 are also thought to play a role in HSC egress from the bone marrow niche. Relatively high S1P in the blood is a major chemoattractant that directs egress of HSC from the bone marrow. Therefore, functional antagonism of the receptor on HSC would limit egress and stabilize newly arrived HSCs following transplantation [76]. This is supported by a study where FTY720 was shown to enhance engraftment of allogeneic bone marrow under reduced intensity conditioning. This effect was not due to reduction of the allogeneic inflammatory response directed to the donor cells as engraftment was enhanced in the absence of allogeneic reaction in syngeneic bone marrow transplantation [77]. Thus, functional antagonism of S1P signaling may enhance engraftment via two mechanisms. Firstly, by increasing homing to the bone marrow in a CXCR4 dependent manner [70] and secondly, by retaining HSC in the bone marrow niche [78].

## 11. Conclusions

The prospect of a cure for leukaemia and non-malignant blood disorders combined with less toxic conditioning regimes has resulted in record numbers of HSCT conducted in recent years [79]. However, there is still a need for new treatment paradigms that control GvHD while maintaining engraftment and GvL. Further understanding of S1P signaling and its role in the various biological processes that occur during and after HSCT, may lead to the utilization of S1P modulators in this setting. Indeed, findings with S1PR1 modulators reviewed here suggest that functional antagonism of S1PR1 has the potential to improve patient outcome by regulating GvH and enhancing engraftment while permitting effective GvL.
